# Horizontal Rafting Plate for Treatment of the Tibial Plateau Fracture

**DOI:** 10.1111/os.12967

**Published:** 2021-05-11

**Authors:** Zhong‐yu Liu, Jin‐li Zhang, Tao Zhang, Qing Cao, Jun‐chao Zhao, En‐qi Li, Qi‐jie Shen, Bao‐cheng Zhao, Yu‐chen Zheng, Yang Chen

**Affiliations:** ^1^ Department of Orthopaedics Tianjin Hospital Tianjin China

**Keywords:** Fixation, Fracture, Horizontal rafting plate, Tibial plateau, Treatment outcome

## Abstract

**Objective:**

This study aimed to investigate the value of a horizontal rafting plate in treating tibial plateau fractures.

**Methods:**

The data of 24 patients in whom a horizontal rafting plate was used to treat a tibial plateau fracture between October 2014 and January 2018 were retrospectively analyzed, including 16 males and 8 females, aged 21–63 years old, with an average of 40 ± 14.68 years. The fractures included 13 in the left knee and 11 in the right knee. The places where the horizontal rafting plate were used included the anterior margin of tibia, anterolateral tibia, and posterolateral tibia. All cases were followed up for 12–24 months, with an average follow‐up of 17.5 ± 5.0 months. At the last follow‐up, the Rasmussen radiological criteria were used to evaluate the effect of fracture reduction and fixation. The knee joint function was evaluated using the Rasmussen functional score. Computed tomography (CT) scanning and three‐dimensional reconstruction were performed preoperatively and postoperatively, with the quality of reduction of the fractured articular surface clarified by the final follow‐up. The flexion and extension abilities of the knee joint were also measured in the postoperative follow‐up.

**Results:**

Preoperative CT scanning showed that the gap of the tibial plateau was 8.00 ± 1.40 (5–24) mm. The heights of the fracture of the articular surface at all three sites during the final follow‐ups were significantly different from the height before the surgery (*P* < 0.05). The vertical distance between the articular line and the highest point of the articular surface after reduction was 0.17 ± 0.05 mm. Anatomic reductions were obtained in 24 patients. The Rasmussen functional score after surgeries was 27.25 ± 0.94 points. Bony union was achieved in all the patients. According to the Rasmussen radiological criteria, the scores during the last follow‐up were as follows: the total score was 13–18 points, with an average of 16.00 ± 1.72 points; the scores were excellent in 17 cases and good in seven cases. Therefore, 100% of results were excellent or good. No infection or fracture nonunion was found.

**Conclusion:**

Using a horizontal plate can be an effective method for treating special types of fractures of the tibial plateau, including the anterior margin and anterolateral and posterolateral tibial plateau, with satisfactory treatment efficacy.

## Graphical abstract

Fixation of the fracture of tibial plateau with the horizontal rafting plate.

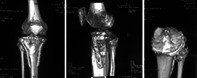

## Introduction

Tibial plateau fracture is an intra‐articular fracture for which the treatment requires not only anatomical reduction but also reliable fixation to maintain the position of the articular surface, allowing patients to receive early function rehabilitation^1^, ^2^. However, inappropriate treatment of the fracture can lead to limited movement, articular degeneration, and long‐term pain^3^, ^4^. The stability of the fixation of the fracture of the tibial plateau improves substantially using an advanced angle‐locking stabilization system and raft‐type fixation for maintaining the reduction of the tibial plateau^5^, ^6^, ^7^. Anatomic locking plates have been developed for fractures medial, lateral, or posterior to the tibial plateau, and therefore various types of fractures can be fixed reliably.

The positions and shapes of the fractures vary greatly due to the differences in the positions of the injuries of the knee joint, as well as the underlying mechanisms, degree of violence, and bone substance involved in the fracture. Some of the fractures are close to the structures of the fibula, patellar tendon, blood vessels, and nerves, which can affect the use of regular plates. For the gap with the margins involved, the reconstruction of the marginal fracture is required to maintain the stability of the knee joint^8^, which cannot be obtained using the currently available plates.

Bermúdez *et al*. ^9^ reported using a horizontal rafting plate for treating a complex tibial plateau fracture. In their study, the conventional reconstruction plate was shaped along the shape of the tibial plateau at the level of the articular rim of the tibial plateau, which was fixed using several screws at different levels. At present, this method is used for the accessory treatment of periarticular fractures, such as fractures of the distal femur, ankle, and proximal humerus^10^. However, for the fracture of the posterolateral tibial plateau, fixation is influenced by the fibula, making fixation very difficult. Jae‐Woo Cho *et al*.^11^ used a horizontal plate for fixing the fracture through the anterolateral approach, which avoided the treatment of such types of fractures *via* the posterior approach and resulted in satisfactory therapeutic efficacy. In 2017, Giordano *et al*.^12^ reported using a “hoop” plate for treating a patient with posterior bicondylar comminuted tibial plateau fractures *via* the lateral approach with a fibular head osteotomy. However, no studies have yet reported the exact sites of the tibial plateau and the clinical efficacy of using the horizontal rafting plate.

This study retrospectively analyzed the data of patients with tibial plateau fractures, who were treated with a horizontal rafting plate so as to investigate the clinical efficacy and application value of this plate for treating tibial plateau fractures at different sites.

## Materials and Methods

### Inclusion and Exclusion Criteria

The inclusion criteria were as follows: (i) patients with fresh tibial plateau fracture who received horizontal rafting plate treatment; (ii) patients aged 20–60 years; and (iii) patients free from severe complications, such as cardiac diseases.

The exclusion criteria were as follows: (i) patients also having knee dislocation; (ii) patients with open fractures; (iii) patients with pathological fractures; (iv) patients with injuries to blood vessels or nerves; (v) patients with severe osteoarthritis with varus or valgus; and (vi) patients with incomplete follow‐up and imaging data or the follow‐up time less than 6 months.

### General Information

From October 2014 to January 2018, this retrospective study included 24 patients (16 males and 8 females) with tibial plateau fractures treated with horizontal rafting plates according to the aforementioned inclusion and exclusion criteria. The study included 16 male and 8 female patients; patients were aged 21–63 years, with an average age of 40 ± 14.68 years. The fracture was in the left knee in 13 patients and in the right knee in 11 patients. The fractures in the patients were categorized into three types according to the sites where the horizontal rafting plate was used for the fractures of the tibial plateau: anterior margin of the tibia in six patients, anterolateral tibia in 13 patients, and posterolateral tibial plateau in five patients. The types of fractures were classified according to the Schatzker criteria, revealing that 14, seven, and three of the patients had type II, type V, and type VI fractures, respectively (Table 1).

**TABLE 1:** Patient demographics

Group	AT (*n* = 6)	ALT (*n* = 13)	PLT (*n* = 5)	*P* value (95% CI)
Age	39.16 ± 14.03	40.84 ± 16.74	30.80 ± 12.13	0.849 (ns)
Sex (male/female)	5/1	8/5	3/2	0.065 (ns)
Classification (II/V/VI)	2/4/0	9/2/2	4/1/0	0.151 (ns)
Follow‐up time	14.83 ± 2.64	17.53 ± 4.75	14.20 ± 2.28	0.951 (ns)

### Standard Protocol Approvals and Patient Consents

All human studies were authorized by the Hospital Ethics Committee and performed in accordance with the ethical standards. Written informed consent was obtained from all patients.

### Treatments of the Patients

*Preoperative preparation*: active symptomatic treatment was initiated after admission, along with the prophylactic use of antibiotics before the surgery. The same group of surgeons performed surgery on all the patients, using lateral tibial plateau locking plate (AO, Germany), microlocking plate (Weigao, China), and L‐type support plate (AO, Germany) for internal fixation.

### Surgical Procedures

Continuous epidural anesthesia or general anesthesia was used on the patients. The position, surgical approach, and internal fixation type were decided according to the types of fractures.

### Fixation of the Anterior Margin of the Tibial Plateau

**Fig. 1:**
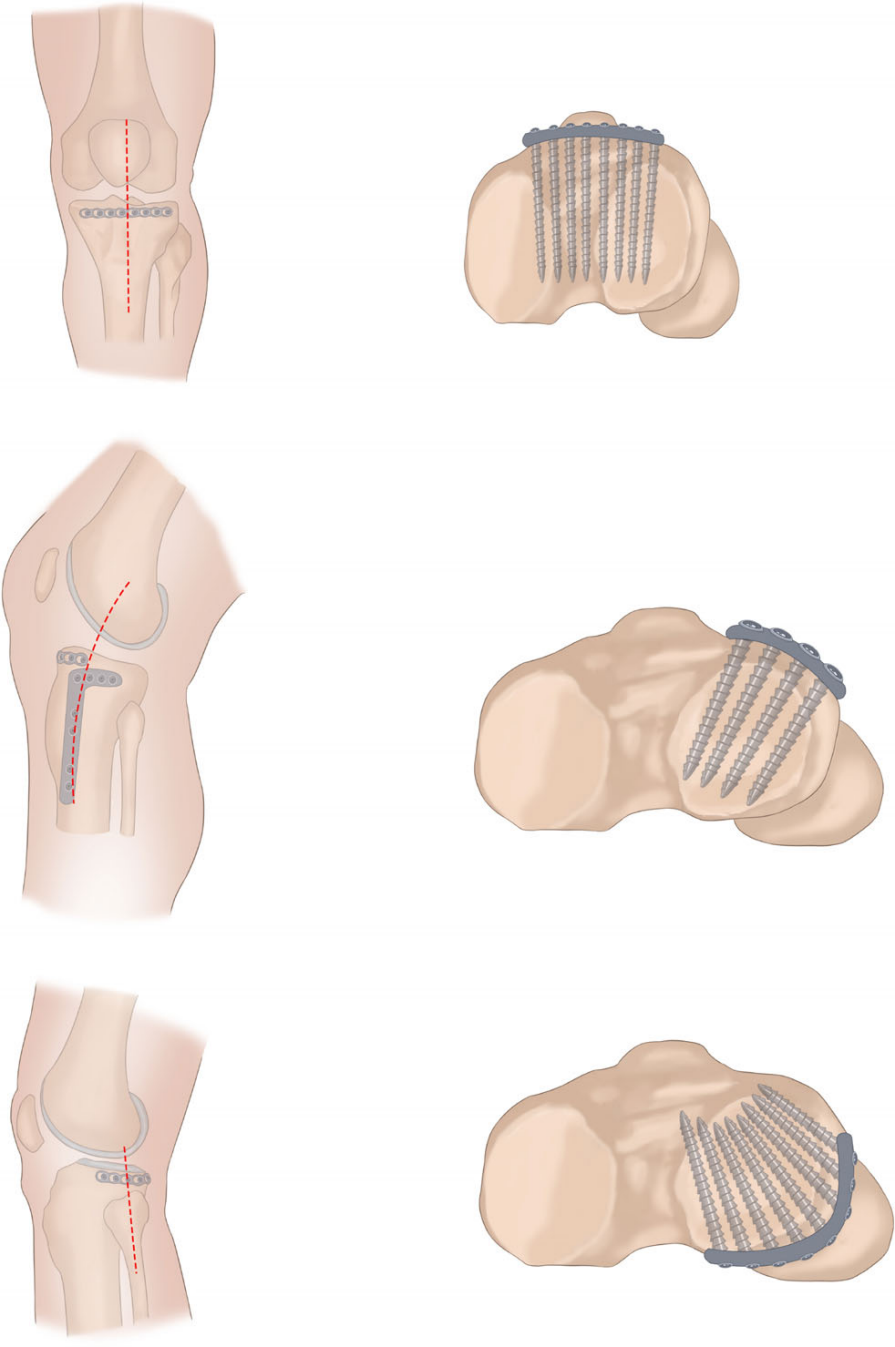
Horizontal plate placement and surgical approach for tibial plateau fractures.

A medial incision and an anterolateral incision were made for the surgery of the patients with a fracture of the anterior tibial margin accompanied by a fracture of the lateral or medial tibial plateau. The medial incision was slightly close to the anterior part, while the skin flap between the lateral and medial incisions was >7 cm. An anterior longitudinal incision was made for separate anterior margin fracture. The surgery was performed *via* the knee anterior median approach for the patients with simple anterior tibial fractures in whom the fracture line was from the anterolateral tibial plateau to behind the patellar tendon. After the fracture of the anterior tibial margin was exposed by pulling the patella from the medial or lateral patellar tendon, a horizontal plate was placed parallel to the articular surface after shaping according to the anterior tibial margin. After one screw was fixed into one end of the plate, a pointed reduction clamp was used to tighten the horizontal rafting plate on the other end to ensure that the formed hoop was wrapped tightly around the anterior cortex (a typical case is shown in Fig. 1).

### Fixation of the Anterolateral Tibial Plateau

The location of the lateral articular fracture was exposed by book‐turning‐like folding of the lateral wall backward along the fracture line on the sagittal plane of the lateral condyle of the tibia. After the reduction of the fracture, the anatomic locking support plate was placed at the proximal lateral tibia, and the locking screws were used at the proximal end of the plate for the “rafting” fixation. The patellar tendon was pulled medially to expose the anterior margin of the lateral tibia, and a pre‐curved plate was placed at that site. The upper margin of the plate was adjacent to the lower margin of the cartilage, the back‐tilt angle of the tibial plateau was measured, and three to five cortical screws with a diameter of 3.5 mm were used accordingly. The direction of the screws was in agreement with the posterior‐tilt angle of the tibial plateau, and it was ensured that the screws did not penetrate through the articular cartilage and into the articular space. Cross distribution was formed by the screws through the anterior margin and the anterolateral margin (a typical case is shown in Fig. 1).

### Fixation of the Posterolateral Tibial Plateau

The microplate was bent and remodeled, which was then used as a horizontal rafting plate. The plate was inserted through the space above the fibular head from the front to the back to allow the wrap around the posterolateral tibial plateau. A long screw was fixed from the front, and then the horizontal rafting plate was tightened to the outline of the bone cortex. Subsequently, a pointed reduction clamp was used to tighten the horizontal rafting plate from the front to ensure that the formed hoop was wrapped tightly around the posterior cortex (a typical case is shown in Fig. 1).

### Postoperative Treatment

After the surgery, the affected limb was immobilized and uplifted, and an elastic bandage was applied for 48 h. The drainage tube was removed 48 h later if the drainage volume was <50 mL. The affected limb of the patients was uplifted, and they were encouraged to early start the isometric contraction of the muscles of the lower limbs. Doppler ultrasound examinations of the veins of bilateral legs on the second day after the surgery showed no signs of thrombosis. In addition, the patients were asked to start the straight leg raising exercise to improve the quadriceps strength. Knee flexion exercise, with the protection of a brace, was started after 1 week of the surgery, and the knee joint of the patients could flex to at least 90° within 2 weeks after the surgery. The weight‐bearing exercise of the toes was performed for 8–12 weeks for the patients, who could achieve full weight bearing 12 weeks later. Knee flexion exercise was also intensified. The prophylactic use of nonsteroid anti‐inflammatory drugs was conducted before the function exercises to alleviate the pain, and a local cold compress with ice bags or ice buckets was used after the function exercises.

### Follow‐up and Treatment Efficacy Assessment

Computed tomography (CT) scanning and three‐dimensional reconstruction were performed preoperatively and postoperatively, with the quality of reduction of the fractured articular surface clarified by the final follow‐up. The data were compared with the preoperative and final follow‐up data. The CT value was measured by two attending orthopedists and an associate chief radiologist independently two times, for which the mean value was calculated.

### Rasmussen Radiological Evaluation

Rasmussen^13^ radiological criteria were used to assess fracture reduction and fixation during the last follow‐up. The assessment included whether the joint surface collapsed, whether the ankle widened, and whether a knee or varus deformity existed. A score of 18 was excellent, 12–17 was good, 6–11 was acceptable, and <6 was poor.

### Rasmussen Functional Evaluation

The Rasmussen functional score ^14^ was used to evaluate the function of patients after surgeries. Five parameters, including severity of pain, deformity on knee extension, walking ability, knee joint stability, and knee joint motion, with a total score of 6 points for each parameter, were included for calculation. The function was classified according to the total score as poor (6–9 points), fair (10–19 points), good (20–26 points), and excellent (≥27 points).

### Statistical Analysis

Statistical analysis was performed using SPSS, version 12.0 (SPSS Inc., Chicago, IL, USA). Postoperative outcomes were retrieved and analyzed. Data are presented as mean ± standard deviation (SD). Age and follow‐up time were compared using analysis of variance; sex and classification were compared using a *χ* ^2^ test. Comparison of the three groups of measurement data was performed by variance analysis, and the difference was statistically significant at *P* < 0.05.

## Results

### Use of Horizontal Rafting Plates at Different Sites

**Fig. 2:**
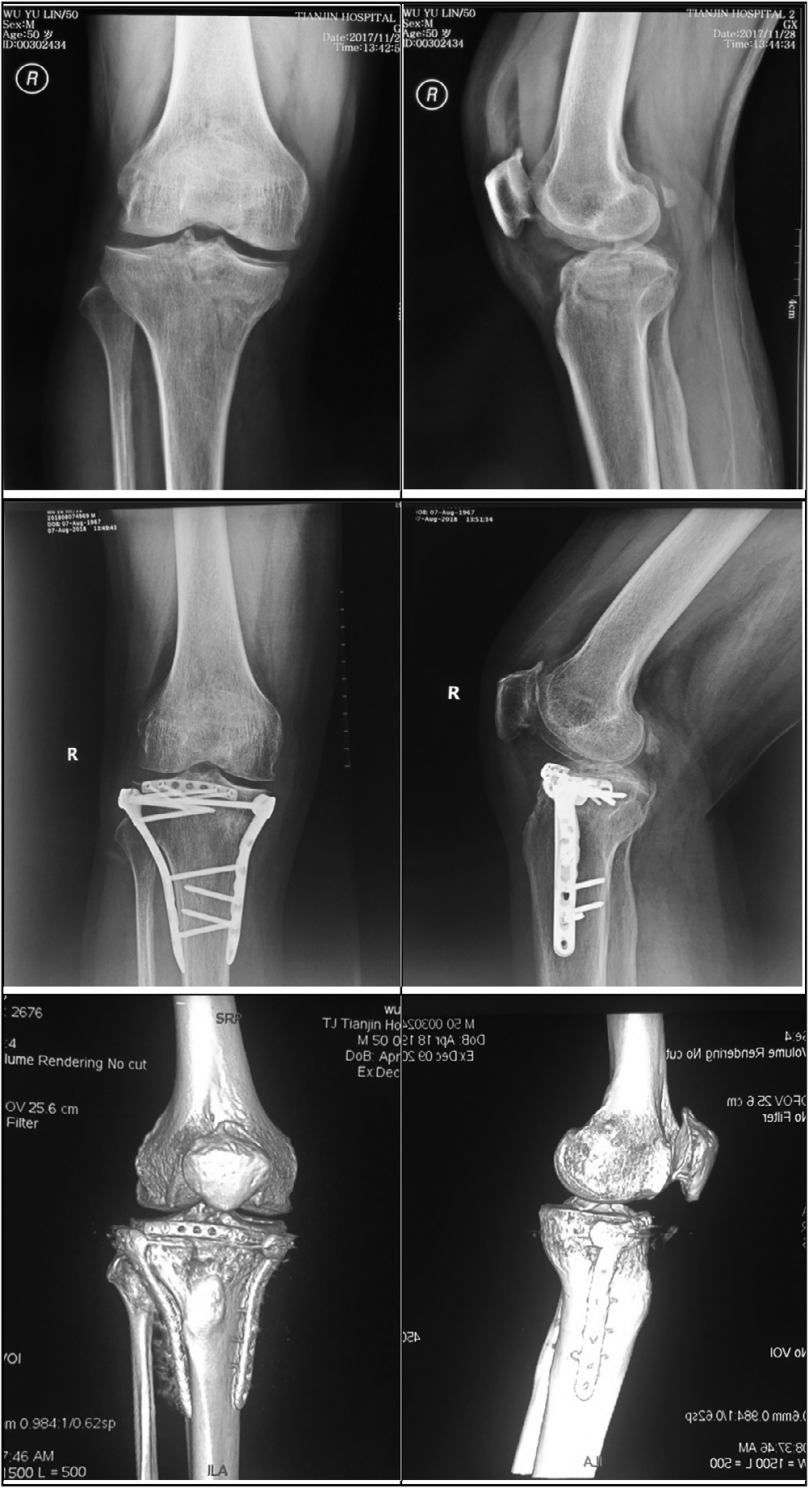
A horizontal rafting plate was used for the fracture of the anterior tibial margin in a 50‐year‐old male patient. The anterior margin of tibial plateau was involved in this tibial plateau fracture. The fracture of the anterior tibial margin was fixed using a horizontal rafting plate.

Horizontal plates for anterior tibia: A seven‐hole plate was used for three patients, an eight‐hole plate for one patient, and a 10‐hole plate for two patients. The plates were placed posterior to the patellar tendon and covered the area enclosed by the patellar tendon (a typical case is shown in Fig. 2).

**Fig. 3:**
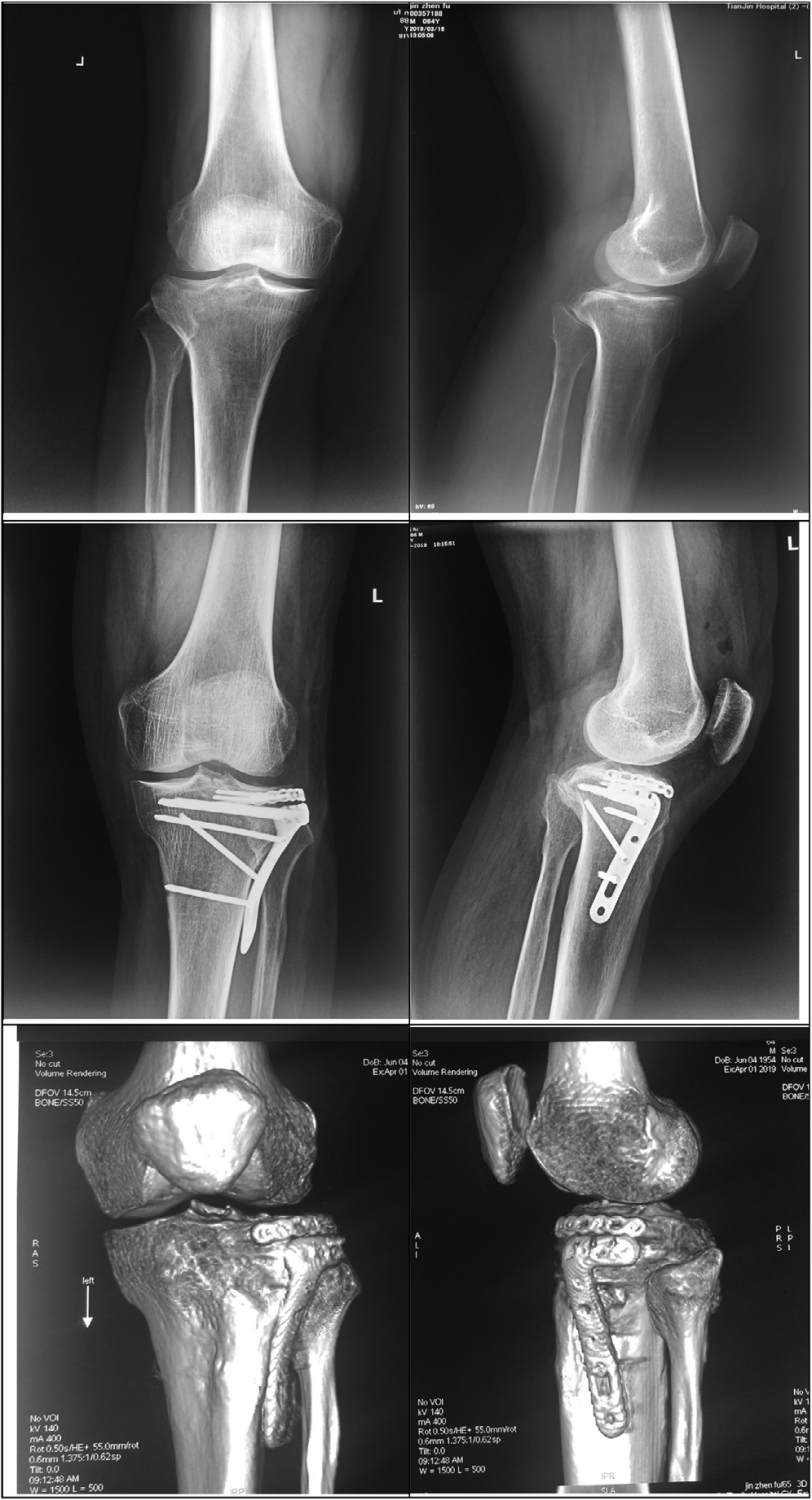
Fixation of the fracture of the anterolateral tibial plateau using a horizontal rafting plate in a 40‐year‐old male patient. A Schatzker type II fracture of the tibial plateau. The anterolateral plateau fracture collapse and splitting existed simultaneously; the articular surface damage was severely accompanied by bone separation and displacement. The anterolateral and posterolateral articular surfaces were reduced and fixed with a locking plate and a horizontal rafting plate.

Horizontal plates for anterolateral tibia: a four‐hole plate was used for nine patients and a five‐hole plate for four patients. An L‐shaped rafting plate was also used at the anterolateral tibia in 10 patients (a typical case is shown in Fig. 3).

**Fig. 4:**
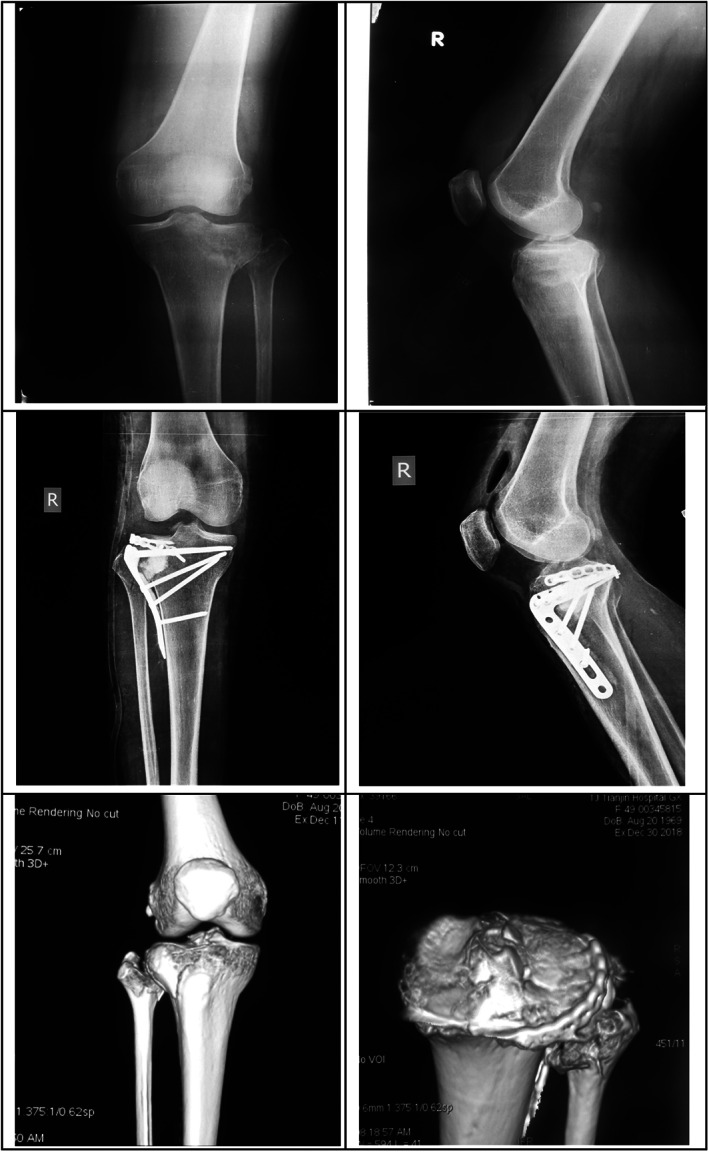
A horizontal rafting plate was used for the posterolateral plateau fracture in a 40‐year‐old male patient. A Schatzker type II fracture of the tibial plateau. The posterolateral platform fracture collapse and splitting existed simultaneously; the articular surface damage was accompanied by bone separation and displacement, and the collapse depth was >10 mm. The anterolateral and posterolateral articular surfaces were reduced and fixed with a locking plate and a horizontal rafting plate. The knee joint function was good after the surgery.

Horizontal plates for posterolateral tibia: a four‐hole plate was used for four patients and a five‐hole plate for one patient. An L‐shaped rafting plate was also used at the anterolateral tibia in four patients (a typical case is shown in Fig. 4).

### Measurement of the Height of Tibial Plateau Fracture

The height of the fracture of the anterolateral articular surface of the tibial plateau was the maximum (24.0 mm) before the surgery, followed by the posterolateral tibial plateau (13.7 mm) and the anterior margin of the tibia (10.3 mm). Preoperative CT scanning showed that the gap of the tibial plateau was 8.00 ± 1.40 (5–24) mm. The CT scanning final follow‐up postoperatively showed that the displacement of the lateral tibial plateau was corrected. The screws through the plate at the anterior margin of the lateral tibial plateau were above the array nails at the proximal end of the plate on the medial and lateral sides of the cancellous bone. The heights of the fracture of the articular surface at all three sites during the final follow‐ups were significantly different from the height before the surgery (*P* < 0.05). After achieving the fracture union and full weight bearing, the CT scanning and three‐dimensional reconstruction showed that the articular surface was smooth and no refracture and displacement occurred. The mean vertical distance between the knee joint line and the highest point of the articular surface was 0.17 ± 0.05 mm, which was significantly different from the distance of reoperation.

### Radiological Evaluation

During the follow‐up period, no reduction loss was observed. The width of the platform and the lower limb force line were normal. No significant abnormalities were found in the width of the platform and the lower limb force line. According to the Rasmussen radiological criteria, the scores during the last follow‐up were as follows: the total score was 13–18 points, with an average of 16.00 ± 1.72 points; the scores were excellent in 17 cases and good in seven cases. Therefore, 100% of results were excellent or good.

### Knee Joint Function Evaluation

The fracture unions of the patients in all the three groups were found after 3 months of the surgery, but no nonunion was found. One patient with a fracture of the anterior tibial margin and another patient with a fracture of the posterolateral tibial plateau had a restricted flexion function of the knee joint after the surgery. The flexion angle was 110° and 115°, respectively. However, the flexion function among the three groups was not significantly different. In addition, the extension function of the patients in all three groups improved evidently, with no significant differences. The average Rasmussen functional score was 27.25 ± 0.94, including six patients with 20–26 points and 18 patients with ≥27 points. Therefore, 100% of results were excellent or good, and the motility levels returned to preoperative levels. Most of the patients could continue their previous work after the surgeries.

### Comparison of Different Fracture Groups

The 24 cases in this group were divided into three groups: AT, ALT, and PLT. In the three groups, there was no significant difference in the follow‐up time, healing time,final follow‐up step, knee movement,and Rasmussen score(Table 2).

**TABLE 2:** Comparison of postoperative outcomes in three subgroups (x ± s)

Groups	Intra‐operative reduction	Follow‐up time (month)	Healing time(week)	Final follow‐up step (mm)	Knee movement(°)	Rasmussen score
AT	Anatomic	14.83 ± 1.08	9.50 ± 0.56	0.18 ± 0.09	133.33 ± 4.77	27.17 ± 0.40
ALT	Anatomic	17.54 ± 1.32	9.69 ± 0.40	0.11 ± 0.55	131.92 ± 1.55	27.46 ± 0.27
PLT	Anatomic	14.20 ± 1.02	10.20 ± 0.73	0.30 ± 0.13	125.00 ± 3.53	26.80 ± 0.37
*P*‐value	‐	0.198	0.720	0.281	0.192	0.418

### Complications

All the surgical incisions in this group healed in one stage, and no complications, such as wound infection, necrosis, and nonunion, occurred. No internal fixation repulsion or looseness was observed. During the final follow‐up, no patients developed symptoms of nonunion or malformation.

One patient in whom the combined anterolateral and posterolateral approach was used showed numbness in the common peroneal nerve innervations and poor ankle joint and toe extension. The symptoms gradually disappeared 2 months after the administration of nutritional peripheral nerve drugs.

## Discussion

### Fixation of the Fracture of the Anterior Tibial Margin Using Horizontal Rafting Plates

The incidence of the fracture of the anterior margin of the tibial plateau is relatively low^15^, ^16^, which is generally caused by hyperextension injuries^15^, ^16^, ^17^. The characteristics of this fracture include the fracture of the anterior margin of the tibial plateau, generally accompanied by the fracture of the articular surface and, sometimes, by injuries of important tendons. Repairing or reconstructing the tendon is generally required to restore the stability of the knee joint. However, such processes can only be used after the reduction and fixation of the fracture^18^, ^19^. All the anterior margin fractures involve a large area and evident comminution or fracture, accompanied by the patellar tendon in the front, which can further increase the difficulties in fixation with conventional plates. To date, only very few studies reported the treatment methods for the fractures of the anterior tibial margin.

The width of a microplate is relatively low, for which the influences on the patellar tendon and surrounding tissues are limited. The use of multiple horizontal screws can result in rafting fixation of the fracture of the anterior tibial margin. The fixation of the anterior tibial margin requires the insertion of the plate inferior to the patellar tendon from one side to the other, thus needing relatively long plates. Therefore, seven‐ to 10‐hole one‐fourth pipe‐type plates were used in this study. If the fracture of the anterior tibial margin was still integrated or with no evident collapse, fixation with the plate from both ends was required. The blocking by the plate could help maintain the positions of the fracture. However, if comminution or collapse was evident in the fracture of the anterior tibial margin, the middle part of the plate should also be fixed with screws to improve the stability of the fracture fragments.

### Fixation of the Fracture of the Anterolateral Tibial Plateau

A fracture of the tibial plateau is relatively common in injuries of the knee joint, especially a lateral condyle fracture, which has the highest incidence^20^, ^21^. Lateral fractures are mainly notching fractures and/or collapsed fractures, involving a large area of the lateral tibial plateau. In addition, such fractures mainly have various comminuted fracture fragments. A tibial plateau fracture is an intra‐articular fracture requiring precise reduction and effective fixation; otherwise, complications such as unstable joint and traumatic osteoarthritis can occur^22^, ^23^, ^24^. Using locking plates for the anterolateral tibial plateau is effective for simple anterolateral fractures. However, the directions and distribution of the locking array nails are limited. Therefore, no effective support and fixation can be provided for massive fractures, and hence the risk of the second fracture of the articular surface after the surgery still exists. Using a horizontal plate can provide supplementary fixation for fracture fragments not fixed using the lateral plateau support locking plates.

The cross‐nail technique is a novel approach introduced in recent years^25^, ^26^. Two screws are knocked in parallelly from outside below the articular cartilage, while two other screws are fixed below the previous two screws parallelly from the right‐front to behind. This parallel cross‐nail technique can help maintain the articular surface after reduction. In this study, two rows of cross‐nail were used to exert the axial anti‐loading effects of the screws. The rafting fixation using three to five cortical screws with a diameter of 3.5 mm from the front to the back through the plate provided sufficient supporting strength and area.

### Fixation of the Fracture of the Posterolateral Tibial Plateau

With the application of plain coronary CT scanning in clinical practice, a fracture of the posterolateral tibial plateau has gained increasing attention from clinical researchers. Surgery for the anatomical reduction of fracture fragments and restoration of the smooth articular surface are the most common treatment for this special type of intra‐articular fracture. However, the structures including the fibular head and the lateral collateral ligament at the posterolateral tibia hinder the exposure of the surgical field. In addition, various blood vessels and nerves at the back of the knee and the muscle fibers of the posterior muscle groups influence the exposure of the fracture. To date, no universally accepted surgical approach and no fixation method are available for treating such fractures. The mainstream treatment method is the support plate fixation *via* the posterior approach or lateral anatomic plate fixation *via* the modified lateral approach^27^, ^28^. However, surgery *via* the lateral approach provides limited surgical space. Further, fixation with a plate through the posterior approach can maintain the reduction of the posterolateral cleavage fracture fragments, but cannot provide support for the posterolateral collapse fracture fragments.

Several studies reported the use of a horizontal plate for fixing posterolateral fractures. Cho *et al*. ^11^ reported the use of the “margin plate” technique, which required the bending and remodeling of a small plate (2.7 mm) for fixing posterolateral tibial plateau fracture fragments. At the distal end of the plate, a regular anatomical locking plate was also used for fixing the proximal tibia. In 2017, Pires *et al*.^10^ reported using a horizontal rafting plate for treating a tibial plateau fracture, with the posterolateral side involved. The surgeries were conducted *via* the fibular neck osteotomy approach to expose the posterolateral plateau. During the surgery, the surgeons were very careful to separate and protect the common peroneal nerve. A bent reconstruction plate was used as the horizontal rafting plate for fixing the posterolateral plateau. In four patients treated in this study, the horizontal rafting plate was used for fixation in patients with the fracture of the posterolateral tibial plateau accompanied by cleavage separation of the fibula. Two patients were treated *via* the fibular osteotomy approach, while the other two patients were treated *via* the Frosch approach. In the Frosch approach, the incision was made from the anterior margin of the biceps femoris muscle toward the anteroinferior side through the fibular head and further extended toward the anterior side. The fracture was exposed in the front and back windows, and then the reduction of the fracture was performed^29^. Previous findings showed that this approach was relatively easy, could expose the full lateral and posterolateral plateaus, and could also allow the treatment of anterolateral and posterolateral fractures. The two windows could help avoid the block of the fibular head and thus avoid fibular head osteotomy.

### Outcomes of the Horizontal Plate for Treating the Tibial Plateau

No special plate has been designed as the horizontal plate yet. Various plates, such as T‐shape plates and one‐third pipe‐type plates, have been remodeled for the horizontal fixation of the tibia. In this study, a one‐fourth pipe‐type plate and microlocking plate were used as the margin plate. Both the one‐fourth plate and microlocking plate had high plasticity; the thickness and width were only 1.5 and 6.5 mm, respectively. Three to four regular screws with a diameter of 2.7 mm were used, and the directions of the screws could be changed freely. The plate was placed posterior to the patellar tendon and at the tibial margin above the fibula. In addition, the screws were closer to the subchondral bones when placed through the plate, thus effectively increasing the supportive effects on the articular surface and providing more space for placing the L‐shape plate.

The measurement of the collapse degree before and after the surgery in this study showed that the positions of the fracture fragments did not change evidently, suggesting that the horizontal plate could maintain the stability of the fracture fragments. Recent biomechanical studies also demonstrated that although the absolute biomechanical strength of horizontal rafting plates was lower than that of posterior support plates, horizontal rafting plates could still provide sufficient fixation, which was enough for early function exercises of the knee joint. The extension ability of the knee joint of the patients in all three groups was well recovered, and the difference among the three groups was not statistically significant. The Rasmussen functional score showed that the patients in all three groups achieved good outcomes, and their motility levels returned to preoperative levels.

### Limitations of the Study

The sample size of the study was relatively small, and the follow‐up time was relatively short. Further large‐sample investigations are still needed to identify other complications. In addition, mechanistic experiments are required to verify the mechanical advantages and reliability of the technique.

### Conclusions

In this study, a one‐fourth pipe‐type plate was used as the horizontal plate for treating fractures of the anterior margin and anterolateral and posterolateral tibial plateaus. The postoperative outcomes suggested that the fixations of fractures were stable, and the treatment efficacies were satisfactory. Using a horizontal plate can be an effective method for treating special types of fractures of the tibial plateau with satisfactory treatment efficacies.
